# Hyperbaric oxygen therapy for ulcerative colitis patients hospitalized for moderate to severe flares (HBOT-UC): study protocol for a multi-center, randomized, double-blind, sham-controlled trial

**DOI:** 10.1186/s13063-025-08932-5

**Published:** 2025-06-22

**Authors:** Lauren B. Bonner, Charlotte Sadler, Peter Lindholm, Denise M. Scholtens, Parambir S. Dulai, on behalf of the HBOT-UC Consortium, Laura E. Kochhar, Gursimran Kochhar, Dana J. Lukin, Michael W. Winter, Corey A. Siegel, Jay C. Buckey, Jenny S. Sauk, Marvin Heyboer, Jeffrey M. Dueker, Ashwin Ananthakrishnan, Udayakumar Navaneethan, Oriana M. Damas, Gerald W. Dryden, Anne G. Tuskey, Kirk B. Russ, Mary Beth Tull, Elizabeth Yan, Yasmin Piña, Libeth Rosas, Chad Rypstra, Brianna Smith, Jamie Heffernan, Gary Toups, M. Hassan Murad, Raymond Shields, Hussam Tabaja, Jayanth Adusumalli, Adina Gutium, Walter Conlan, Richard Viglione, Aaron Cohn, Jamie B. Wabich, Ganesh Aswath, Gerald H. Markovitz, Juan C. Jimenez, Xiaoxiao Yin, Rasha Abed, Danny Acevedo, Mark Ajalat, Juan O. Bravo, Stephanie Ioannou, Angelina Meza-Suarez, Monica Perez, Oreana Teran, Niurka Colina, Michael Mijares, Matthew P. Kelly, Benjamin D. Rogers, Matthew Scanlon, Ahmad AlhajHusain, Brittany J Behar

**Affiliations:** 1 Department of Preventive Medicine, Division of Biostatistics and Informatics, Northwestern University, Chicago, IL USA; 2 Division of Hyperbaric Medicine, Department of Emergency Medicine, University of California San Diego, La Jolla, San Diego, CA USA; 3 Division of Gastroenterology and Hepatology, Northwestern University, Chicago, IL USA

**Keywords:** Ulcerative colitis, Hyperbaric oxygen therapy, Randomized trial, Study protocol

## Abstract

**Background:**

Chronic intestinal hypoxia and accompanying mucosal inflammation is a hallmark of ulcerative colitis. Hyperbaric oxygen therapy involves breathing 100% oxygen under increased atmospheric pressure to increase tissue oxygenation. It reduces systemic and local inflammation and up-regulates hypoxia response pathways, making it an attractive therapeutic option. In this trial we aim to confirm the treatment benefits of hyperbaric oxygen therapy for hospitalized ulcerative colitis patients and assess the long-term durability of treatment effect.

**Methods:**

This prospective, double-masked, multicenter, 1:1 randomized, sham-controlled trial will enroll 126 participants with known or newly diagnosed ulcerative colitis hospitalized for an acute moderate to severe flare. Participants will be randomized to either hyperbaric oxygen therapy with steroids or sham air with steroids. The trial will involve a 5-day intervention period followed by a 12-month observational period with a 90-day standard of care visit and 12-month telephone visit. The primary outcome measure is clinical response defined as complete resolution of rectal bleeding and improvement in stool frequency, without need for in-hospital biologics, small molecules, or colectomy by study day 5. Secondary endpoints include additional key patient-reported outcomes and histo-endoscopic measures of disease activity.

**Discussion:**

Novel and effective treatments are needed for this population to optimize disease outcomes while minimizing treatment-related risks. Demonstrating the ability of hyperbaric oxygen therapy to improve clinical response to steroids and avoid in-hospital rescue therapy has the potential to change the management of hospitalized ulcerative colitis flares.

**Trial registration:**

ClinicalTrials.gov NCT05987852. Registered on August 14, 2023.

## Administrative information

Note: the numbers in curly brackets in this protocol refer to SPIRIT checklist item numbers. The order of the items has been modified to group similar items (see http://www.equator-network.org/reporting-guidelines/spirit-2013-statement-defining-standard-protocol-items-for-clinical-trials/).

**Table:** 

Title {1}	Hyperbaric oxygen therapy for ulcerative colitis patients hospitalized for moderate to severe flares (HBOT-UC): study protocol for a multi-center, randomized, double-blind, sham-controlled trial
Trial registration {2a and 2b}.	ClinicalTrials.gov: NCT05987852
Protocol version {3}	Protocol Version 4.0; November 22, 2024
Funding {4}	The National Institute of Diabetes and Digestive and Kidney Diseases (NIDDK) U01DK134321
Author details {5a}	Lauren B. Bonner^1^, Charlotte Sadler^2^, Peter Lindholm^2^, Denise M. Scholtens^1^, Parambir S. Dulai^3^ 1. Department of Preventive Medicine, Division of Biostatistics and Informatics, Northwestern University, Chicago, IL, USA 2. Division of Hyperbaric Medicine, Department of Emergency Medicine, University of California San Diego, La Jolla, CA, USA 3. Division of Gastroenterology and Hepatology, Northwestern University, Chicago, IL, USA
Name and contact information for the trial sponsor {5b}	Trial Sponsor: Northwestern University Contact: Parambir S. Dulai, MD Address: 259 E Erie St. Chicago, IL 60611 Email: Parambir.dulai@northwestern.edu
Role of sponsor {5c}	HBOT-UC is an investigator-initiated trial. Thus, the sponsor is directly involved with the initiation, design, and execution of the trial. The funder (NIDDK) is involved in monitoring the ongoing progress of the trial. They will not have any role in the execution, analysis, interpretation of data, or reporting of results.

## Introduction

### Background and rationale {6a}

Ulcerative colitis (UC)-related hospitalizations pose a significant burden to patients and healthcare systems. Annually in the USA, there has been a significant increase in UC-related hospitalizations and UC-related hospitalizations [1]. The need for hospitalization represents a major event in a UC patient’s disease course, and patients remain at a significantly increased risk for re-hospitalization, colectomy, and morbidity and mortality for up to 5 years [2–7]. Despite the burden of UC-related hospitalizations, current therapies are unsafe and ineffective for this patient population. Colectomy is curative for UC; however, it has a 5% post-operative mortality risk when done emergently in the hospital and a substantial impact on long-term morbidity and quality of life [8, 9]. Goals of medical therapy for hospitalized UC patients are therefore to avoid colectomy while minimizing disease- and treatment-related complications. High-dose intravenous steroid therapy is the mainstay of medical therapy, but over half of patients are refractory to intravenous steroids and require second line rescue therapy with biologics or small molecules [10, 11]. These immunosuppressive drugs are associated with serious infections, malignancy, and considerable cost [12, 13]. New treatment options are therefore needed to optimize disease outcomes while minimizing treatment-related risks.

Hyperbaric oxygen therapy (HBOT) targets mechanisms central to the pathogenesis of UC and mucosal integrity. Mechanisms thought to play a role in UC include (1) a dysregulated response to tissue hypoxia with altered barrier function; (2) an aberrant host response to gut flora with shifts in microbial composition; and (3) recruitment and activation of neutrophils and pathogenic T cells in the bowel leading to cytokine production and inflammation [14–20]. HBOT involves breathing 100% oxygen under pressure to increase plasma and tissue oxygen levels. The high levels of oxygen created by HBOT have been shown to produce a variety of downstream effects that persist well after the participant leaves the chamber which include (1) reductions in inflammation and inflammatory cytokine production, (2) interfering with neutrophil trafficking to target tissues, (3) shifts in host-microbiome metabolism and microbial composition, (4) stabilization of hypoxia response pathways (HIF-1α, HO-1), and (5) increased growth factor synthesis and stem cell migration to improve wound healing [21–27]. HBOT may therefore offer a promising new treatment as it targets several of the mechanisms involved in UC pathogenesis [26].

A meta-analysis across 17 studies of patients with Crohn’s disease (*n* = 286) or UC (*n* = 327) provided evidence of safety and potential efficacy for treating inflammatory bowel disease (IBD) patients [26]. IBD patients treated with HBOT had an overall response rate of 86%. Analyses limited to studies with endoscopic follow-up indicated an overall response rate for UC patients of 100%. HBOT was also well tolerated by IBD patients, with adverse event rates (10 per 10,000 HBOT sessions) and serious adverse events (6 per 10,000 sessions) being rare. Separate Phase 2 A and Phase 2B multicenter, randomized, sham-controlled trials were conducted to provide preliminary evidence in support of HBOT for UC. In the Phase 2 A trial, the treatment effect benefit was primarily seen in the first 3 days of therapy, with maximal benefit achieved by day 5 (day 5 clinical remission: 50% HBOT vs 0% Sham, *p* = 0.04) [28]. A subsequent Phase 2B trial demonstrated that five HBOT sessions (compared to three) were needed to achieve maximal clinical benefit [29].

Given the minimal risks associated with HBOT and preliminary evidence of clinical improvement in UC patients with HBOT, a larger trial is warranted to confirm efficacy.

### Objectives {7}

The primary objective of the HBOT-UC trial is to confirm the ability of HBOT to improve clinical response to steroids, while avoiding need for biologics, small molecules, or colectomy in-hospital, in UC patients hospitalized with moderate to severe flares. The secondary objective is to assess the long-term durability of treatment effect over 12 months, and to further investigate the effects of HBOT on additional key patient reported outcomes and histo-endoscopic measures of disease activity. Additional exploratory objectives are to assess treatment effect heterogeneity by subgroups of interest and to assess differences in intestinal ultrasound measures of disease activity. This will serve as a basis for explaining or supporting findings from primary analyses and for further investigating whether treatment effects may vary for different subgroups.

### Trial design {8}

This two-arm parallel group, multicenter, double-masked trial will randomize 126 participants 1:1 to either HBOT + steroids or sham hyperbaric air + steroids. The study will consist of a 5-day intervention period, followed by a 12-month observational follow-up period, with a standard of care visit at day 90 and a 12-month telephone follow-up. All analyses are based on a superiority framework.

## Methods: participants, interventions, and outcomes

### Study setting {9}

Patients hospitalized for a UC flare will be screened for inclusion and exclusion criteria prior to or upon admission to the hospital. Participating academic centers or community-based gastroenterology practices with in-patient facilities must have access to hyperbaric oxygen chambers and be able to deliver the active and sham interventions. A list of study sites can be found in the ClinicalTrials.gov record.

### Eligibility criteria {10}

All inclusion and exclusion criteria rely on information available from standard of care testing and medical records for an UC patient hospitalized for an acute flare, and no specific testing will be required for the trial to assess for inclusion/exclusion criteria. Eligibility criteria are included in Table 1.

**Table 1:** Inclusion and exclusion criteria for HBOT-UC

Inclusion criteria	• Known or newly diagnosed moderate to severe UC (as defined by the Mayo score of 6–12; confirmed by clinical, endoscopic, and/or histopathological evidence prior to screening as per standard of care) who require hospitalization for an acute flare with the additional following criteria: • Mayo endoscopic sub-score ≥ 2 (moderate to severe) • Mayo rectal bleeding sub-score ≥ 2 (moderate to severe) • Mayo stool frequency sub-score ≥ 2 (moderate to severe) • Minimum disease extent of > 15 cm from the anal verge • Consented and able to receive first HBOT session within first 48 h of initiation of intravenous steroids • Age 18–85 • Able to fully participate in all aspects of the trial • Agreement to not participate in another trial for the duration of the active intervention period of the trial
Exclusion criteria	• Received HBOT either as part of standard of care or through a clinical trial • Complication requiring urgent surgical intervention (in the opinion of the investigators) • Requirement for new start of a biologic or small molecule during the hospitalization prior to randomization and/or anticipated requirement for rescue medical or surgical therapy within 48 h of randomization • Toxic megacolon, suspected or impending toxic megacolon, or colonic dilation on imaging • Inability to receive intravenous steroids per the dosing schedule as specified in the protocol due to intolerance, risks, and/or participant preferences • Participants who have historically failed or been exposed to 4 or more classes of advanced therapeutic options • Known or suspected diagnosis of Crohn’s colitis, indeterminate colitis, ischemic colitis, radiation colitis, diverticular disease associated with colitis, microscopic colitis or infectious colitis (Clostridium difficile, cytomegalovirus, any other pathogenic illness felt by the investigator to be the source of colitis) • Received any investigational drug within 30 days prior to consent • Clinically significant cardiac, renal, neurological, endocrine, respiratory or hepatic impairment that increases the risk for HBOT toxicity in the opinion of the investigator • Women who are pregnant or nursing. Women with childbearing potential will be required to use effective birth control if not surgically sterile or postmenopausal for ≥ 2 years for the duration of the active intervention period (5 days) • Unwillingness to complete course of HBOT or to return for the remainder of HBOT (or sham) sessions if they are discharged prior to day 5

### Who will take informed consent? {26a}

Each prospective participant will be given a full explanation of the study and allowed to read the Informed Consent Form (ICF). Once the site investigator or designee is assured that the participant understands the implications of participating in the study, the participant will be asked to give consent to participate in the study by signing the ICF. The informed consent process will be briefly described in the source documents for each participant. One original ICF will be signed by the participant. A copy of the signed ICF will be provided to the participant. The original signed ICF will be maintained in the participant’s medical records at the clinical center.

### Additional consent provisions for collection and use of participant data and biological specimens {26b}

As part of participation in the study, participants are asked to provide biological specimens (stool and tissue samples) and give permission to use video recordings of colonoscopies and/or flexible sigmoidoscopies. The ICF includes details that participant data and biospecimens could be used for future research studies without additional informed consent.

## Interventions

### Explanation for the choice of comparators {6b}

The population of interest is at an increased risk for needing rescue medical and/or surgical therapy in-hospital to prevent disease-related complications, morbidity, and/or mortality. The decision to initiate rescue therapy is based on provider assessments. In an unblinded trial design, there is a risk that the provider may unnecessarily delay initiation of rescue therapy awaiting a response to hyperbaric oxygen. To avoid the potential for provider bias in making decisions for rescue medical and/or surgical therapy, a double-blind study design is required with a sham hyperbaric intervention. As described in detail below, standardized sham treatment protocols for hyperbaric therapy were implemented based on relevant chamber types and with input from international experts [28, 30].

### Intervention description {11a}

HBOT: Participants enrolled in the active intervention group receiving HBOT will undergo compression to a maximum pressure of 2.4 atmospheres absolute (ATA; 100% oxygen). Participants will receive 100% oxygen for 30-min periods for up to three 30-min periods, with up to two 5–10-min air breaks (breathing room air at 2.4 ATA) between periods [28, 29]. This will be done for both the monoplace and multiplace chambers for the active intervention group.

Monoplace Sham: This control arm will breathe 21% oxygen (room air) and undergo compression to a maximum pressure of 1.34 ATA at the beginning of each treatment. The chamber will then be decompressed from 1.34 to 1.2 ATA. Participants will then be maintained at 1.2 ATA breathing 21% oxygen (room air) for up to three 30-min periods, with up to two 5–10-min air breaks (breathing room air at 1.2 ATA), between periods. All air given to the participants in the Sham arm is to be given as room air. Multiplace sham: This control arm will undergo compression to a maximum pressure of 2.4 ATA, but 21% oxygen instead of 100% oxygen will be administered. Participants will receive 21% oxygen (room air) for 30-min periods for up to three 30-min periods, with up to two 5–10-min air breaks (breathing room air at 2.4 ATA) between periods. To avoid a risk for decompression sickness in this multiplace sham arm, we will follow a protocol developed for inside attendees of these multiplace hyperbaric chambers. In this protocol, the sham treated participants would breathe 100% oxygen for up to 15 min prior to ascent identical to the inside attendant of the chamber. Given all participants are wearing hoods and the oxygen concentration is managed by the attendant, this would allow for maintenance of blinding for all participants in the chamber if multiple participants were enrolled simultaneously, with some enrolled in the sham arm and some in the active intervention arm. Prior published experiences with over 24,000 exposures observed a risk of decompression sickness of 0.00021% with this protocol [31].

Standard of Care Treatment: All participants enrolled will follow standard of care treatment for a hospitalized UC flare, which will include daily intravenous steroids (60 mg Solumedrol once a day or 100 mg Hydrocortisone three times a day). If they meet criteria for discharge prior to completing day 5 of the study period, they will be discharged on an oral regimen of steroids.

Post-Discharge Treatment: No standardized recommendations, protocols, or guidelines exist for the management of UC patients in the post-discharge period following an acute severe flare or during the 12 months following discharge. Outpatient societal recommendations do exist, however, for the treatment of moderate to severe UC flares and management of biologics or small molecule inhibitors [32, 33]. Providers will be advised to follow these standard-of-care outpatient recommendations when managing UC participants. Complete standardization of all post-discharge care was deemed to be not possible due to individual participant preferences, and local payor approvals and/or access variability across institutions which would influence decisions on the choice of therapies. Suggested care algorithms may be updated over time if recommendations change and/or newer therapies become available.

### Criteria for discontinuing or modifying allocated interventions {11b}

A participant may be withdrawn from the intervention if (1) the participant experiences an adverse event and continued participant poses an unacceptable risk to the participant’s health, (2) the participant wishes to stop the intervention, or (3) there is a significant protocol deviation and continued participant poses an unacceptable risk to the participant’s health. Participants who are withdrawn from the intervention will remain in the study and continue to complete follow-up assessments. Participants who were withdrawn from the study due to colectomy will be treated as completing the study and endpoint evaluation will incorporate the colectomy as described in the statistical analysis plan. If a participant is withdrawn from the study and is no longer willing to complete follow-up visits, the reason for withdrawal will be documented in the database. Assessments will be completed at the time of withdrawal to capture available data on patient reported outcomes, medication use, laboratory results, and disease progression to the extent possible.

### Strategies to improve adherence to interventions {11c}

Given that the active intervention period is only 5 days and these participants are hospitalized for the duration of the intervention, we anticipate high adherence to the intervention. Participants will be allowed to leave the chamber for non-adverse-related reasons (such as diarrhea). Participants who have not completed a minimum of 60 min at maximum pressure will be asked to attempt repeat treatment.

To ensure the intervention is delivered as intended, a Manual of Procedures includes detailed steps for delivery of the intervention, including a checklist and guidance for communication with the participant. An electronic case report form will document any departures from the intervention protocol and reasons for any incomplete sessions.

### Relevant concomitant care permitted or prohibited during the trial {11d}

Participants are not permitted to participate in another trial for the duration of the active intervention period (5 days). Patients are allowed to receive rescue medical and/or surgical therapy prior to study day 5 if deemed to be a non-responder based on standardized definitions and/or if felt to be in the best clinical interest of the participant by the treating provider to avoid disease-related complications.

### Provisions for post-trial care {30}

If a participant is injured as a result of the study intervention or from procedures completed for the purposes of this study, the study sponsor will not pay for medical expenses.

### Outcomes {12}

The primary outcome is clinical response, a binary indicator measured by complete resolution of rectal bleeding (Mayo rectal bleeding sub-score of 0) and improvement in stool frequency (at least 1 point reduction in Mayo stool frequency), without the need for biologics, small molecules, or colectomy assessed at day 5. If a participant is discharged prior to day 5, any biologics or small molecules taken after discharge do not contribute to the endpoint definition. Additionally, participants who are discharged before day 5 with complete resolution of rectal bleeding and improvement in stool frequency, without the need for biologics, small molecules, or colectomy will be considered as clinical responders regardless of day 5 assessments. Data will be summarized as the proportion meeting the clinical response definition.

Key secondary outcomes include (1) clinical response, a continuous measure of the sum of Mayo rectal bleeding and stool frequency sub-scores summarized as the mean at day 3; (2) C-reactive protein (CRP), a continuous measure summarized as the mean at day 3; and (3) endoscopic response, a continuous measure defined by the Ulcerative Colitis Endoscopic Index of Severity (UCEIS) summarized as the mean at day 5. Additional secondary endpoints include (1) steroid-free, colectomy-free, clinical remission (Full Mayo score ≤ 2, with no sub-score > 1), a binary indicator summarized as the proportion at day 90; (2) endoscopic improvement, a binary indicator defined by a Mayo endoscopic sub-score of 0 or 1 summarized as the proportion at day 90; (3) endoscopic remission, a binary indicator defined by a Mayo endoscopic sub-score of 0 summarized as the proportion at day 90; (4) mucosal healing, a binary indicator defined by a Mayo endoscopic sub-score of 0 or 1 and a Geboes histology score ≤ 2 summarized as the proportion at day 90; (5) clinical remission, a binary indicator defined by a full Mayo score ≤ 2 with no sub-score > 1 summarized as the proportion at day 5; (6) need for colectomy, a binary indicator summarized as the proportion at day 90 and (7) the proportion at 12 months; (8) Re-hospitalization for an ulcerative colitis flare, a binary indicator summarized as the proportion at 12 months; (9) Urgency Numeric Rating Scale (NRS), a continuous measure summarized as the mean at day 3; and (10) any serious infection or serious adverse event, a binary indicator summarized as the proportion at day 5. Of note, continuous outcomes violating normality assumptions may be summarized with medians.

Despite the lack of formal consensus for primary endpoints in clinical trials for UC patients hospitalized for moderate-severe flares, prior clinical trials in this population have used stool frequency and rectal bleeding measures [32, 33]. Similarly, evidence-based reviews have suggested these metrics for determining hospital discharge [34, 35]. Therefore, we have chosen resolution of rectal bleeding (Mayo rectal bleeding sub-score of 0) and improvement in stool frequency (at least a 1-point reduction in Mayo stool frequency sub-score) as our primary endpoint for clinical response. Additional rationale for the primary endpoint is detailed elsewhere [36]. Recognizing the importance of endoscopic and histologic measures of disease activity and FDA guidance for outpatient trials, endoscopic and histologic improvement during hospitalization and follow-up have been incorporated as secondary outcomes. Colectomy and hospitalizations were noted in the SPIRT consensus created by the International Organization for Inflammatory Bowel Disease (IOIBD) to be key midterm complications to assess for in disease modification trials [37]. CRP is encouraged to be collected by the FDA [38] and the IOIBD consensus statements also note this as an important secondary outcome [39–41].

### Participant timeline {13}

**Fig. 1:**
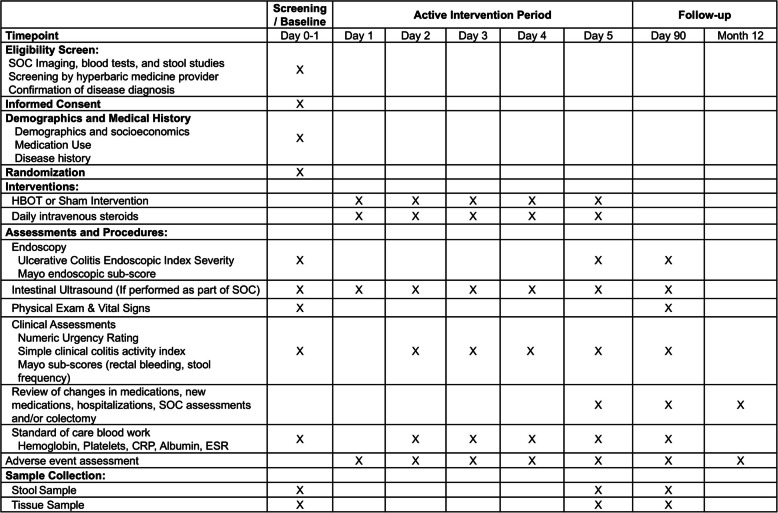
HBOT-UC study schematic

Participants are expected to participate in the study for approximately 1 year. Most of the study burden will occur during the first 5 days. Following screening, informed consent, and randomization, the participant will receive 5 consecutive days of the intervention (HBOT or sham air). A standard of care follow-up visit will occur at approximately 90 days, and a telephone call with the participant will occur at approximately 12 months. Additional details are provided in the study diagram (Fig. 1).

### Sample size {14}

Sample size estimates were based on the primary aim of detecting a clinically meaningful increase in the frequency of clinical response/remission in the HBOT arm compared to the control arm. Specifically, we estimate that our primary outcome will be achieved in 30% of hyperbaric oxygen treated patients and in 10% of sham hyperbaric air treated patients. Based on combined phase 2 A and 2B trial data, we found 11 of 22 (50%, 95% CI: 28–72%) patients who received the HBOT achieved normalization of rectal bleeding and improvement in stool frequency [28, 29]. Given the small sample sizes of these trials, we have chosen the lower bound of this confidence interval (30%) for the point estimate of the primary outcome in the HBOT arm. Outpatient moderate-severe ulcerative colitis trials have observed a 10% placebo response rate for resolution of rectal bleeding by day 5 of active therapy. Although our population being recruited is sicker and the anticipated placebo/sham response rate would be lower than seen in outpatient trials, we have chosen a conservative estimate of 10% for the primary outcome in sham treated patients. For 80% power, with 1:1 randomization and a 5% two-sided significance level, we estimate we will need 63 patients per arm to detect an absolute difference in clinical remission of 20% (30% in HBOT arm vs. 10% in Sham), based on the standard sample size calculation formula for a two-sample test of proportions.

### Recruitment {15}

All patients admitted to the participating sites with a diagnosis of ulcerative colitis will be identified by a member of the team through admissions, emergency department, or gastroenterology consulting services within the respective hospitals. As these patients require specialty care, the consultation of a gastroenterologist is considered standard of care when ulcerative colitis patients are admitted for an acute flare.

## Assignment of interventions: allocation

### Sequence generation {16a}

Participants will be randomized via permuted block randomization, with blocks of varying sizes, stratified by bio-naïve or bio-exposed status. The treatment arm randomization sequence will be created by the Northwestern University Data Analysis and Coordinating Center (NUDACC), serving as the Data Coordinating Center (DCC), and uploaded to the electronic study database. The block sizes will not be disclosed, to ensure concealment.

### Concealment mechanism {16b}

The unmasked hyperbaric oxygen staff or unmasked study coordinator will only be able to access the randomization form in the database once a participant has been screened and consented, preventing the study team from knowing the next randomization assignment until the participant is randomized.

### Implementation {16c}

Once a patient has been screened and consented, the hyperbaric oxygen staff member will access the randomization assignment in the database.

## Assignment of interventions: blinding

### Who will be blinded {17a}

Masking will be maintained for participants, hospital and gastroenterology research staff, and treating providers. The only unmasked personnel from sites will be the hyperbaric oxygen department/center staff, who require knowledge of the treatment assignment to ensure appropriate interventions are delivered and participants can be monitored and managed appropriately based on the delivered intervention. The DCC includes both masked and unmasked project management staff and statisticians. To prevent accidental contamination, a dedicated unblinded study e-mail account was created for sites to communicate with unblinded DCC team members. Access to randomization assignment and intervention information is restricted to only the unmasked team. Additionally, days 0, 5 and 90 Mayo endoscopic sub-score and Geboes histology score will be done by masked centralized readers.

### Procedure for unblinding if needed {17b}

A participant’s treatment assignment may be unmasked by the unblinded hyperbaric medicine provider if the participant is experiencing symptoms of decompression sickness and knowledge of the treatment assignment is required to treat the adverse event.

## Data collection and management

### Plans for assessment and collection of outcomes {18a}

Demographic information, medical history, ulcerative colitis disease history, and medication use will be collected from the participant and/or medical record. Stool studies to rule out infectious etiologies of presentation will be conducted during screening. A physical examination at baseline and day 90 will assess for any clinical abnormalities and vital signs will be recorded. Daily standard of care blood work will include complete blood count, CRP, erythrocyte sedimentation rate (ESR), and a basic metabolic panel including albumin.

During the 5-day intervention period, data will be collected by the hyperbaric oxygen therapy staff regarding session completion and duration at pressure. Daily intravenous steroids and venous thromboembolism prophylaxis use will be recorded.

An endoscopy with biopsies for histopathology will be conducted at screening, day 5, and day 90. The Mayo endoscopic sub-score and the UCEIS [42] will be ascertained by local treating providers and/or study investigators. Available videos from procedures will be captured for re-review centrally. Stool samples will be collected at screening/baseline, day 5, and day 90. Pre-packaged collection kits with instructions will be provided to sites and utilized to ensure appropriate collection technique for stool.

Clinical assessments will include the Mayo score, Urgency Numeric Rating Scale (NRS), and the simple clinical colitis activity index (SCCAI). The Mayo score is a validated instrument to measure disease activity and is comprised of 4 components: rectal bleeding, stool frequency, physician assessment, and endoscopy appearance [43]. Each component score ranges from 0 to 3, with a total score ranging from 0 to 12 (mild disease activity: 3–5, moderate disease activity: 6–10, severe disease activity: 11–12). The partial Mayo score (excluding the endoscopic measure) will be assessed daily through the active intervention period, with the total score being assessed at screening, day 5, and day 90 to correspond with the timing of endoscopies. The NRS scale is a newly developed, validated, and reliable score designed to assess the severity of bowel urgency [44]. The urgency and nocturnal bowel movement frequency sub-scores of the SCCAI are a validated tool designed to assess colitis exacerbations [45]. The NRS and SCCAI will be collected daily through the active intervention period and at day 90.

Review of changes in medications, new medications or hospitalizations, and adverse event assessments will be conducted throughout the 12-month follow-up. Data from additional endoscopies and intestinal ultrasounds, performed as part of standard of care, within the follow-up period will be collected.

To promote data quality and rigor, site staff will be trained on the protocol and data entry. A data entry vignette will be provided to ensure staff are familiar with entering data into the REDCap project. Additionally, NUDACC will run regular data status and quality reports to identify missing data and potential data entry errors. NUDACC will also perform remote source document verification for one participant per site per year for key data. Data collection forms will be made available upon request.

### Plans to promote participant retention and complete follow-up {18b}

Retention of participants for the study intervention period is anticipated to be 100%, as these participants are hospitalized for their acute flares and are therefore available for daily hyperbaric oxygen therapy sessions. If a participant is discharged prior to day 5, the participant will receive remuneration for returning to complete the daily HBOT sessions. Retention during the 12-month follow-up study period will be optimized by provider contact through routine care given how high risk these participants are for disease relapse and/or progression to biologics or colectomy, and coordinator contact from the local sites. Additional remuneration will be provided for the day 90 standard of care visit and 12-month phone call.

### Data management {19}

NUDACC has designed and built a dedicated REDCap [46, 47] database for the trial. The database mirrors corresponding paper case report forms, and sites have the option of entering data directly into the electronic database or first recording data on paper case report forms. Database access will be audited, and role-based security will be used to provide seamless access and integration to functions such as data entry, data download, and generation of reports. NUDACC will determine and regulate access to data resources through the assignment of security roles, and Data Access Groups will allow for restrictions and control on data entry occurring at each study site.

Centralized training will provide demonstration and ensure comprehension among all relevant study staff at participating sites. Validations for data entry will be programmed wherever possible to ensure consistency and accuracy. NUDACC will also generate a series of data quality assessment scripts as described above. When inconsistencies, discrepancies, or other quality assessment issues are observed, the Data Resolution Workflow module in REDCap will be used to generate queries on specific fields. The Data Resolution Workflow will provide an audit trail for every field for which any discrepancies are noted.

### Confidentiality {27}

Participant privacy and confidentiality will be protected by various security procedures. A study document linking the participant to the study ID will be securely stored at the corresponding clinical center and not transmitted to the DCC. Any paper documents used for data collection will be securely stored within the respective Gastroenterology department of each center for a length of time determined by local site policies. All electronic case report form data will be stored in the secure REDCap database or on secure servers. NUDACC will keep a full copy of the dataset for at least 5 years, and data will be stored indefinitely at the NIDDK Central Repository, as described in {31c}. The site Principal Investigators will ensure confidentiality and masking of study arm.

### Plans for collection, laboratory evaluation, and storage of biological specimens for genetic or molecular analysis in this trial/future use {33}

Pre-packaged collection kits with instructions will be provided to sites and utilized to ensure appropriate collection technique for stool. These will include pre-made bar codes for scanning and storage upon return. All samples will be kept locally in a − 80 °C freezer at each site using de-identified study ID numbers and accession numbers, and batch shipped on dry ice to the central coordination center.

Dedicated research mucosal biopsy specimens will be obtained from participants. We will also rely on the standard of care histopathology specimens being collected during lower endoscopies. Standard of care biopsies at these lower endoscopies will undergo standard pathologic evaluation of colon biopsies and storage of specimens per the Department of Pathology standard protocols at each institution. Additional slides will be created from the standard of care pathology specimens for spatial transcriptomics analyses and centralized histology scoring.

Human biospecimens collected from the trial which remain at the end of the study will be transferred to the central repository. All personal identifiers will be removed from the specimens and the accompanying data prior to transfer. NUDACC will facilitate the transfer of study data, study documentation, study forms, and linkage files (as applicable). All future researchers who utilize those samples must do so under a Usage Agreement which stipulates that researchers will only conduct research consistent with the subjects’ informed consent and will not attempt to identify any individuals.

## Statistical methods

### Statistical methods for primary and secondary outcomes {20a}

Primary analyses will be based on an intention-to-treat principle, whereby all patients will be analyzed as randomized. Descriptive statistics will summarize all baseline variables by arm, and there will be no formal hypothesis testing for comparison of baseline characteristics between treatment arms. Standardized differences in covariates may be examined to evaluate covariate balance across groups. Residual diagnostics will be examined, and relevant assumptions will be assessed using graphical display and statistical testing where appropriate. In situations where modeling assumptions are in question, nonparametric methods, transformations, or inclusion of higher-order terms may be applied.

Primary analyses will employ a logistic regression model to estimate the odds of clinical response in the treatment arm compared to the control arm, adjusting for the stratification factor (bio-naïve or bio-exposed). The odds ratio and 95% confidence interval will be reported with the corresponding p-value.

A linear mixed model will be used for key secondary continuous endpoints (clinical response and CRP), incorporating data from daily assessments (day 2 and day 3). Specifically, the mixed model will include fixed effects for arm (HBOT vs. sham), timepoint (visit day), the interaction between arm at timepoint (to allow for different differences between arms at each visit), corresponding baseline measure, and the stratification factor. For both endpoints, the hypothesis of interest will be based on the difference in score at day 3. For UCEIS (not assessed daily), a linear model will be used to estimate the difference in mean UCEIS score at day 5 between arms adjusting for baseline UCEIS and the stratification factor. In primary analyses, participants with an early colectomy preventing assessment of UCEIS at day 5 will be excluded from the analysis. A correction for multiple testing of the three key secondary endpoints will be incorporated using the Holm method.

Additional secondary analyses will incorporate similar models for all additional binary outcomes. Participants receiving a colectomy prior to the time point of analysis will be considered as ‘non responders’ (e.g., no clinical remission or no endoscopic improvement). A linear mixed model will be used for the continuous endpoint NRS, incorporating data from daily assessments as described above in key secondary analyses. As in primary and key secondary analyses, models will include adjustment for the stratification factor. All additional secondary analyses will be assessed using a two-sided type I error rate of 0.05.

### Interim analyses {21b}

To provide confidence that our trial will be well powered at completion, we will perform an interim analysis for re-estimation of the sample size at approximately 50% information (*N* ≈ 63). The sample size re-estimation will be based on a conditional power approach as described by Mehta and Pocock [48]. The proposed approach allows for incorporation of an unblinded sample size re-estimation without inflating type I error. If the estimate of conditional power at interim analysis is within the pre-defined promising zone, we will consider an increase in the total sample size. If conditional power is outside the promising zone, we will continue with the planned sample size. The promising zone defines a range at which interim conditional power is below the unconditional power targeted during study design, but high enough that increasing the sample size at or below the maximum feasible sample size will recover the targeted power. Increasing the sample size only if the conditional power falls within this range preserves type I error for final analyses. Detailed results of the interim analysis will be kept securely with the unblinded DCC team. There will be no formal stopping rules for efficacy or safety. Adverse events (AEs) and serious adverse events (SAEs) will be recorded on dedicated case report forms and will be reviewed on an ongoing basis by the DSMB. The trial may be stopped early for safety at the recommendation of the DSMB.

### Methods for additional analyses (e.g., subgroup analyses) {20b}

Exploratory analyses will also assess heterogeneity of treatment effects by planned sub-groups. Specifically, separate models will be built and the interaction term between treatment and each individual sub-group (i.e., age group (> 65 vs 18–65), sex, prior hospitalization, prior biologic and/or small molecule exposure) will be included in the logistic regression model, described in primary analyses. Depending on the distribution of categorical variables, binary variables may also be considered.

Planned sub-groups:

- Age (> 65 years versus 18–65 years)
- Gender (male versus female)
- Prior hospitalization for an ulcerative colitis flare (yes versus no)
- Prior biologic and/or small molecule exposure (yes versus no)
- Number of prior biologic and/or small molecule exposures (0, 1, 2, 3 or more)
- Type of biologic and/or small molecule being used at time of hospitalization
- Steroid dependent and/or resistant at the time of hospitalization (yes versus no)
- Baseline endoscopic severity (Mayo endoscopic subscore of 2 versus 3)
- Baseline C-reactive protein (< 5 mg/L versus ≥ 5 mg/L)
- Steroid type during hospitalization (methylprednisolone versus hydrocortisone)
- Chamber type during trial (monoplace versus multiplace)
- Day 3 response status during trial (day 3 responder versus non-responder)

### Methods in analysis to handle protocol non-adherence and any statistical methods to handle missing data {20c}

A sensitivity analysis will be conducted on the per-protocol sample including participants who (1) completed at least 3 full, consecutive intervention sessions, where a “full” session is defined as at least 60 min under pressure; (2) do not have an “early” colectomy, defined as a colectomy on or before day 3; and (3) do not have protocol deviations for unacceptable treatment adjustments through day 3. Results will be used to complement the primary planned analyses based on the intention-to-treat sample.

Given the short time frame and nature of hospitalization, we do not expect any attrition for the primary endpoint. For secondary analyses beyond day 5, we do anticipate larger dropout. In the event of large amounts of missing data (i.e., more than 10%), multiple imputation analyses will be explored. Mechanisms of missing data will be examined, with an assessment of variability in missing rate by participant characteristics. These summarizations will inform potential biases resulting from missing data. If appropriate, multiple imputation methods will be considered in sensitivity analyses. Results will be compared across approaches to assess the effect of missing data, robustness to various model specifications, and validity of specified models.

### Plans to give access to the full protocol, participant-level data, and statistical code {31c}

Participant level data will be transferred to the NIDDK central repository at completion of the study. Statistical code will be available upon request.

## Oversight and monitoring

### Composition of the coordinating centre and trial steering committee {5d}

The HBOT-UC study is governed by committees designed to oversee specific aspects of study conduct. The Executive Committee will include NIDDK representatives, the Clinical Coordinating Center PI, and the Data Coordinating Center PI. The Executive Committee is tasked with setting the overall direction for the study, including reviewing ongoing study progress, establishing the study subcommittees, and appointing subcommittee chairs. The Steering Committee will include approximately 2–4 site PIs, representing the clinical centers, and all members of the Executive Committee. The Steering Committee will be charged with implementing, coordinating, and managing the trial. Subcommittees will be formed to (1) monitor recruitment and retention, (2) track amount and quality of samples transferred to biorepository and NIDDK central repository, (3) review revisions of the Manual of Procedures and training materials, (4) review authorship and dissemination of results in presentation and publications, (5) review all ancillary study proposals, and (6) ensure compliance with NIDDK Data Sharing Policies and National Institutes of Health Public Access Policy. A non-significant risk designation for our trial has been obtained from the FDA. We will follow abbreviated reporting requirements, but an investigational device exemption is not required.

### Composition of the data monitoring committee, its role and reporting structure {21a}

The HBOT-UC study has an independent DSMB appointed by NIDDK to oversee study participant safety. The DSMB will receive reports on study progress and safety as well as data quality. Participants in both arms will be assessed for serious and for non-serious adverse events throughout the study period. The DSMB will meet on a semi-annual basis to monitor cumulative safety data and data quality.

### Adverse event reporting and harms {22}

All adverse events (AEs) (not including expected ulcerative colitis-related events (UCREs)) experienced by the participant between the signing of the ICF and end of study visit will be reported. All events meeting criteria for a Serious Adverse Event (SAE) will be documented in the database. Expected UCREs (e.g., diarrhea, blood in stool, or colectomy) will not be documented as AEs unless the event is of greater intensity, frequency, or duration than expected or the investigator believes there is a reasonable possibility the event was related to the intervention. However, these UCREs will be systematically assessed through various other data collection instruments including the Mayo score. The relationship of the AE to the study intervention and/or other study treatments will be assessed separately by independent medical monitors. Any event meeting the criteria of an unanticipated adverse device effect will be reported to the FDA, sIRB, and NIDDK.

Safety data will be presented by treatment groups, and events will be categorized according to the Common Terminology Criteria for Adverse Events (CTCAE) coding system. Safety analysis will consist of describing AEs, clinical laboratory abnormalities, or changes in vital signs that occur in each treatment group over the follow-up period. Trial publications will include descriptive statistics of all AEs occurring during the study period, which will be provided for each treatment group. Additionally, AEs will be summarized by the number and percentage of subjects experiencing any AEs, any severe AEs, any treatment-related AEs, any SAEs, any treatment-related SAEs, and any AEs leading to study treatment interruption.

### Frequency and plans for auditing trial conduct {23}

Remote monitoring visits will occur approximately once per site per year to perform remote source document verification for one randomly selected participant. NUDACC, in collaboration with the HBOT Steering Committee and NIDDK, may schedule on-site monitoring visits throughout the study as needed. These visits will occur at the discretion of the study team and may be linked to site-specific concerns. These visits may be conducted to follow up on training needs and action items, or if remote source documentation or central monitoring reveals continuing or serious noncompliance with data entry and/or study procedures. Additionally, the sponsor or sIRB may visit the site to conduct an audit of the study in accordance with regulatory guidelines.

### Plans for communicating important protocol amendments to relevant parties (e.g., trial participants, ethical committees) {25}

All protocol modifications will undergo review by the sIRB and will be approved before being released to the sites. Changes will be communicated formally by the DCC and discussed at HBOT Steering Committee meetings, study-wide investigator meetings, and coordination staff committee meetings. Changes will also be communicated to the DSMB and modifications will be made to the ClinicalTrials.gov record.

Any new important information that is discovered during the study and which may influence a participant’s willingness to continue participation will be provided to the participant. Additionally, reconsent will be obtained following any major protocol and ICF modifications, as appropriate.

### Dissemination plans {31a}

Trial results will be disseminated through publication of results in peer-reviewed journals as well as dissemination at national or international conferences.

## Discussion

Demonstrating efficacy of HBOT, to optimize early response to steroids, has the potential to shift the paradigm in management of hospitalized UC flares. Given that HBOT is already widely available nationally and internationally, the intervention would be readily implementable in practice. Strengths of this trial include the pragmatic trial design increasing efficiency and generalizability; the inclusion of geographically diverse centers drawing from a demographically diverse population; and the relevance of key outcomes to patients and clinicians. Operational challenges include the short window in which potential participants must be identified, screened, enrolled, and randomized. The trial requires close communication between the gastroenterology and HBOT teams, to ensure a participant can receive the first HBOT session within 48 h of initiating intravenous steroids. The trial is also relying on a clinical device that is used in routine care for other indications, and accessibility of the chambers or technical support for chamber operations during off-hours may pose a challenge throughout the study period. Other challenges include evolutions in biologic and/or small molecule treatment landscape for moderate-severe UC in the outpatient setting which may pose challenges to recruitment.

## Patient Involvement

Patients were involved in the design of this research through the Participant Centered Care Committee. This committee reviewed patient centricity and feasibility for the study design and endpoints.

## Trial status

The first participant was enrolled in March 2024, and we anticipate enrollment completion by March 2027. At the time of publication, the trial is utilizing protocol version 4.0; November 22, 2024.

## Data Availability

Participant level data and biospecimens will be transferred to the NIDDK central repository at completion of the study. All future researchers who utilize these data and samples must do so under a Usage Agreement.

## Abbreviations

AE: Adverse event
ATA: Atmospheres absolute
CRP: C-reactive protein
DCC: Data Coordinating Center
DSMB: Data and Safety Monitoring Board
ESR: Erythrocyte sedimentation rate
FDA: Food and Drug Administration
HBOT: Hyperbaric oxygen therapy
IBD: Inflammatory bowel diseases
ICF: Informed consent form
IOIBD: International Organization for Inflammatory Bowel Disease
NIDDK: National Institute of Diabetes and Digestive and Kidney Disorders
NRS: Numeric Rating Scale
NUDACC: Northwestern University Data Analysis and Coordinating Center
SAE: Serious adverse event
sIRB: Single Institutional Review Board
SCCAI: Simple Clinical Colitis Activity Index
UC: Ulcerative colitis
UCEIS: Ulcerative Colitis Endoscopic Index of Severity
UCRE: Ulcerative colitis-related event
